# Impact of Malaria on Hematological Parameters in People Living with HIV/AIDS Attending the Laquintinie Hospital in Douala, Cameroon

**DOI:** 10.1371/journal.pone.0040553

**Published:** 2012-07-10

**Authors:** Gervais Gouana Tchinda, Julius Atashili, Eric A. Achidi, Henri L. Kamga, Anna L. Njunda, Peter M. Ndumbe

**Affiliations:** 1 Department of Medical Laboratory Sciences, Faculty of Health Sciences, University of Buea, Buea, Cameroon; 2 Department of Public Health and Hygiene, Faculty of Health Sciences, University of Buea, Buea, Cameroon; 3 Department of Biomedical Sciences, Faculty of Health Sciences, University of Buea, Buea, Cameroon; Johns Hopkins Bloomberg School of Public Health, United States of America

## Abstract

**Background:**

People living with HIV/AIDS (PLWHA) frequently have abnormal blood counts including anemia, leucopenia and thrombocytopenia. The role of infection with plasmodia on these hematological parameters in PLWHA is not well known. In this study we compared selected hematological parameters between malaria positive and negative PLWHA.

**Methods:**

We conducted a cross-sectional study of PLWHA attending the Douala Laquintinie hospital. After obtaining consent, demographic and clinical data were obtained via a standardized questionnaire. Blood samples collected for hematological assays were run using an automated full blood counter. Malaria parasitaemia was determined by blood smear microscopy.

**Results:**

A total of 238 adult PLWHA were enrolled, 48.3% of who were on antiretroviral therapy and 24.8% of whom had malaria parasitaemia. The respective mean (±SD) of hemoglobin level, RBC count, WBC count, platelet count, lymphocyte count and CD4+ T cell counts in malaria co-infected patients versus non-infected patients were: 10.8(±1.9) g/dl versus 11.4(±2.0)g/dl; 3,745,254(±793,353) cells/µl versus 3,888,966(±648,195) cells/µl; 4,403(±1,534) cells/µl versus 4,920(±1,922) cells/µl; 216,051(±93,884) cells/µl versus 226,792(±98,664) cells/µl; 1,846(±711) cells/µl versus 2,052(±845) cells/µl and 245(±195) cells/µl versus 301(±211) cells/µl. All these means were not statistically significantly different from each other.

**Conclusion:**

There was no significant difference in studied hematological parameters between malaria positive and negative PLWHA. These data suggest little or no impact of malaria infection. Hematological anomalies in PLWHA in this area need not be necessarily attributed to malaria. These need to be further investigated to identify and treat other potential causes.

## Introduction

Malaria and HIV are two important causes of morbidity and mortality in Africa. Over two-thirds of people living with HIV/AIDS (PLWHA) are found in sub-Saharan, a region in which malaria is endemic [1]. HIV infection is the leading cause of mortality amongst adults aged 15–59 years in the region [2]. On the other hand, malaria is a life-threatening infection with an estimated 216 million cases and 655,000 deaths per year globally, with approximately 91% of these deaths occurring in sub-Saharan Africa [3]. Recent studies suggest that mortality due to malaria may have been previously underestimated: it is now estimated that, worldwide, close to 1.2 million people died of malaria in 2010, with 1.1milllion of these occurring in Africa [4].

Although malaria and HIV infections are highly prevalent and remarkably overlapping in Sub-Saharan Africa, the extent and implications of their interactions are still not completely understood [5]. Initial descriptions suggested no more than clinical coexistence [6]. More recently, studies in regions of stable malaria transmission have noted higher parasite rates in co-infections than in mono infections of malaria or HIV; increased incidence of severe malaria in co-infected HIV-positive individuals, and increased mother-to-child transmission of HIV in HIV-positive mothers with placental malaria [7], [8].Also, some of these studies reported a reduction in the following blood parameters: hemoglobin concentrations, erythrocyte sedimentation rates (ESR), packed cell volume (PCV), platelet count, differential lymphocyte counts and CD4+ T cell counts in co-infected patients compared to patients with single infections of either type [5], [9], [10]. These hematological changes could be understood in light of the mechanisms by which malaria and HIV individually impact hematological parameters: HIV can result in a general myelossupressive effect, while malaria could impact hematological parameters through its hemolytic effect on red blood cells as well as its immuno-inflammatory effects on white blood and platelet cell counts.

In Cameroon, an estimated 610,000 people were living with HIV in 2009, with an adult prevalence of 5.3% [1], while the annual adult malaria incidence was estimated at over a million [3]. A recent study conducted in Douala (Cameroon) reported a high prevalence rate of co-infections of 29.4% with anemia as the most prominent hematological anomaly [11].

This study aimed at assessing the impact of co-infection with malaria on some hematological parameters in HIV-infected people. Such information is expected to help health care practitioners improve on current diagnostic, therapeutic and follow up guidelines of HIV-infected patients presenting with hematological abnormalities.

## Methods

### Study Design and Participants

This was a cross-sectional study carried out between the months of April and June 2010 at the “day care center” of Laquintinie Hospital, Douala, Cameroon.

Douala is situated in the tropical rainforest zone of the Congo basin. As the economic capital of Cameroon, Douala attracts people of all social backgrounds seeking employment, besides business people and tourists. The area has rainy and dry seasons, running from mid-march to October and from November to mid-march respectively. The town has several swampy sites, stagnant water pools around houses in unpopular quarters especially during the rainy season, poor waste disposal in a constantly increasing population; all factors that support breeding of female Anopheles mosquitoes, the vectors of the malaria parasite. Malaria in Douala and most of Cameroon is endemic, with high transmission. Malaria is predominantly due to *Plasmodium falciparum*. Douala therefore, was an ideal site to investigate HIV/malaria co-infection.

Included in this study were non-pregnant adult people living with HIV/AIDS (PLWHA) presenting at the Laquintinie Hospital for routine follow up and/or treatment. After providing written consent, participants were interviewed using a standardized structured questionnaire. Blood samples were then collected for thick and thin smear microscopy as well as a standard full blood count. Each participant’s medical records were also reviewed to consider past and present underlying opportunistic infections reported, especially those usually affecting blood cells.

### Laboratory Assessments

Using sterile plastic syringes, 3 mls of venous blood was aseptically collected into sterile EDTA-vacutainer tubes for laboratory investigations. Thick and thin films were prepared from a portion of blood for each participant’s blood sample within 30 minutes of collection. After fixing thin smears with absolute methanol, smears were stained using 10% Giemsa for 10 minutes and read by microscopy [12]. Each thick and thin film was assessed by two microscopists.

Another portion of blood was used for full blood counts using the Hema Screen18 automated full blood counter (Hospitex Diagnostics, Sesto Fiorentino, Italy). CD4+ T cells were enumerated using the Partec CyFlow® automated cell counter (Partec GmbH, Munster, Germany). Malaria parasitaemia was measured as parasites per microliter based on the measured white blood cell counts (WBC).

### Statistical Analysis

Data collected were entered into EPI-INFO version 3.5.1 software (CDC/WHO, Atlanta, USA) and analyzed using STATA version 10 (Statacorps, Texas, USA).Two sample Student t-tests were used to assess differences in means between malaria positive and negative participants. To account for potential confounding, multivariate analyses were conducted using multiple linear regression models. Each model had the specific hematological parameter being analyzed (RBC, Hgb, WBC, Lymphocyte count, CD4+ count or platelet count) as dependent variable; and malaria parasitaemia (positive versus negative), age, gender, CD4+ cell count and antiretroviral therapy as independent variables. Pearson correlation coefficients were also used to quantify the relationship between malaria parasitaemia and each of red blood cell count, hemoglobin levels, white blood cell counts, lymphocyte counts, CD4+ cell counts and platelet counts, among malaria positive participants. To assess if there were differences (in the impact of malaria parasitaemia on haematological parameters) by potential modifiers (CD4 count, antiretroviral therapy, gender and age), we created product interaction terms between parasitaemia and each of the potential modifiers in multivariate linear regression models. None of these interactions were statistically significant. The statistical significance level was set at 0.05.

## Results

A total of 238 non-pregnant adults fulfilling the inclusion criteria voluntarily participated in the study. Of these 161 (67.6%) were females. Study participants’ characteristics are summarized in Table 1.

**Table 1:** Characteristics of 238 participants included in study of role of malaria parasitaemia in hematological parameters of people living with HIV/AIDS in Douala, Cameroon.

		Total		Plasmodium-positive (n = 59)	Plasmodium-negative (n = 179)	P-value
Characteristic	Level	n	%	%	%	
Gender	Female	161	67.6	76	65	0.10
	Male	77	32.4	24	35	
Age (years)	20–29	29	12.2	14	12	0.91
	30–39	91	38.2	41	37	
	40–49	67	28.2	27	28	
	50+	51	21.4	19	22	
Marital status	Single	85	35.7	0	3	0.40
	Married	99	41.6	42	41	
	Divorced	6	2.5	41	34	
	Widow(er)	48	20.2	17	21	
CD4+ count	<200	99	41.8	53	38	
	200+	138	58.2	47	62	
Antiretroviral therapy	No	123	51.7	53	51	0.88
	Yes	115	48.3	47	49	
Type of Antiretroviral therapy**	Zidovudine	55	23.1	29	21	0.23
	Stavudine	24	10.1	3	12	0.05
	Lamivudine	99	41.6	37	43	0.44
	Nevirapine	44	18.5	15	20	0.46
	Efavirenz	50	21.0	20	21	0.88
Self-reported use of any malaria prevention method*	No	55	76.9	27	22	0.40
	Yes	183	23.1	73	78	

* including use of bednets or insecticides or other environmental measures such as avoiding pools and bushes around living environment.

** Categories are not mutually exclusive.

In terms of HIV-related comorbidities, 42 patients had a history of pulmonary tuberculosis while three participants each had a history of toxoplasmosis, Pneumocystis pneumonia, oral candidiasis, and HSV disease; two had a history of cryptococcal meningitis and one had a history of oesophageal candidiasis. The CD4+ cell count was <200 in 99 (41.8%) of participants and 48.3% of all participants were on antiretroviral therapy.

Plasmodia were detected in 24.8% of participants. Parasitaemia ranged from 30–26,550 with a median of 3,875 (IQR: 1,120–7,660) parasites/µL.

Hematological parameters in malaria positive and negative participants are compared in Table 2. With differences of less than 10%, red blood cell counts and platelet counts were very similar between malaria positive and negative participants (p-value>0.05). Hemoglobin levels, white blood cell counts, lymphocyte and CD+ cell counts were slightly lower in malaria co-infected participants although the difference did not achieve statistical significance. These findings were similar in both bivariate and multivariate analysis (adjusting for age, gender, CD4+ cell count and antiretroviral therapy).

**Table 2:** Comparison of hematological parameters between plasmodium-positive and plasmodium-negative HIV-infected participants in Douala, Cameroon.

Hematological parameter	Plasmodium-positive(n = 59)	Plasmodium-negative (n = 179)X	Mean difference	P-value	Adjusted Mean difference*	P-value
	Mean ± SD	Mean ± SD	(SE)		(SE)	
RBC count (cells/µl)	3, 745,254±793,353	3, 888,966±648,195	−143,713 (103,090)	0.165	−92,021 (99,694)	0.36
Hemoglobin (g/dl)	10.8±1.9	11.4±2.0	−0.6 (0.29)	0.056	−0.22 (0.24)	0.36
WBC count (cells/µl)	4403±1534	4920±1922	−517 (275)	0.060	−350 (276)	0.21
Lymphocyte counts(cells/µl)	1845±710	2051±844	−206 (122)	0.093	−95 (113)	0.40
CD4+ counts (cells/µl)	244±194	301±211	−56 (31)	0.072	−55 (29)	0.06
Platelet count (cells/µl)	216,050±93,884	226,791±98664	−10,740 (14,638)	0.464	−11,509 (14,881)	0.440

* adjusting for Age, gender, CD4+ cell count and whether on antiretroviral therapy or not.

Amongst the 59 participants in whom plasmodia were detected, the plasmodium parasitaemia levels were not significantly correlated with each of red blood cell count, hemoglobin levels, white blood cell counts, lymphocyte counts, CD4+ cell counts and platelet counts (Table 3).

**Table 3:** Correlation of between malaria parasitaemia and hematological parameters in 59 plasmodium positive HIV-infected participants in Douala, Cameroon.

Hematological parameter	Correlation coefficient	P-value
RBC count (cells/µl)	−0.03	0.796
Hemoglobin (g/dl)	−0.15	0.251
WBC count (cells/µl)	0.13	0.333
Lymphocyte counts (cells/µl)	0.02	0.888
CD4+ lymphocyte counts (cells/µl)	−0.17	0.212
Platelet count (cells/µl)	−0.15	0.252

## Discussion

Hematological anomalies are considered a hallmark of malaria, and reported to be most pronounced in *Plasmodium falciparum* infections [13]. In this study of PLWHAs, the mean values of hematological parameters appeared lower in participants having malaria parasitaemia when compared to those who were diagnosed negative. However, the statistical insignificance of these differences suggested that though HIV and malaria have each been reported to cause myelosuppression by Bashawri *et al*., [14] and Suresh *et al*., [10] respectively, hematological anomalies such as anemia and/or cytopenia may not automatically worsen when combined infections occur.

The mean red blood cell count and hemoglobin concentrations were not significantly lower in malaria parasitaemia positive participants than in malaria negative participants (P>0.05). The insignificant difference in hemoglobin concentrations between these groups was contrary to previous findings by Erhabor *et al*., [9] and Nkuo-Akenji *et al*., [11] who found malaria to be significantly associated to anemia in HIV/AIDS infected persons.

The mean lymphocyte and total white cell counts in malaria positive participants were not significantly different from those in malaria negative participants (P>0.05). These observations agreed with previous findings by Erhabor *et al*., [9], meaning that malaria in HIV-infection is not associated to leucopenia.

The mean platelet count in malaria positive participants was not significantly different from that in malaria negative participants (P>0.05). This was not in accordance with the previous finding that mean platelet count in plasmodium parasitized individuals HIV/AIDS patients was significantly lower as compared to non-parasitized controls [9].

Lastly, the mean CD4^+^ cell count was not significantly lower in malaria positive participants (245±195) when compared to malaria negative participants (301±211). This was in accordance with findings reported by Audu *et al*., [15] that neither malaria nor tuberculosis has an effect on CD4 counts. However, this finding was not in agreement with previous findings that CD4^+^T lymphocytes decline temporarily during malaria episodes in HIV-infected and uninfected persons [16] and that repeated malaria infection are associated with more rapid decline in CD4^+^ T lymphocytes over time [17].

Multiple factors could contribute to haematological parameters in people living with HIV/AIDS. These include nutrition and intake of nutrients such as iron, vitamin B12, folic acid, the use of antiretrovirals some of which may impact haematological parameters, opportunistic infections that may modify the immune response and thus modify WBCs and lymphocyte counts as well as therapy for these other opportunistic infections, latent undetected neoplasia and malaria. The relative role of each of these factors could vary from population to population. If the lack of a difference with respect to malaria parasitaemia in this population was confirmed, this would suggest that other factors may have a predominant role. In the meantime, clinicians need to be aware to provide a wider assessment for PLWHA who present with an anomaly in their haematological parameters.

While studying a population living in endemic area, use of an accurate quantification for parasitaemia and the use of multivariate analysis controlling for age, gender and antiretroviral use were strengths of this study, the sample size did not allow for a simultaneous adjustment accounting for many other factors such as cotrimoxazole use, and other comorbidities (which were rare, not systematically screened for and thus potentially misclassified). The relatively small sample size of malaria co-infected people living with HIV could also have resulted in a lack of statistical significance in the observed differences, thus these findings may need to be confirmed in a study with a larger number of malaria positive participants. Also, the cross-sectional design precludes from inferring a causal role (or lack thereof) of malaria parasitaemia on haematological parameters.

In conclusion, this comparison of mean red blood cell counts, mean hemoglobin concentrations, mean lymphocyte count, mean platelet counts, total white cell count and CD4 counts between malaria parasitaemia positive and negative people living with HIV/AIDS in Douala suggests no significant differences. These findings indicate that malaria may not be the primary cause or correlate of hematological disorders in these patients. As such findings of hematological anomalies should not be assumed to be due to malaria – a more specific etiologic investigation is needed to establish the likely etiology. Longitudinal studies, in large cohorts, investigating the medium and short term evolution of these hematological anomalies and the relationship with malaria and malaria treatment are also needed to more accurately aid the investigation and management of hematological anomalies in people living with HIV/AIDS.
